# Omega 3 fatty acids increase the chemo-sensitivity of B-CLL-derived cell lines EHEB and MEC-2 and of B-PLL-derived cell line JVM-2 to anti-cancer drugs doxorubicin, vincristine and fludarabine

**DOI:** 10.1186/1476-511X-12-36

**Published:** 2013-03-16

**Authors:** Johannes F Fahrmann, W Elaine Hardman

**Affiliations:** 1 Department of Biochemistry and Microbiology, Marshall University School of Medicine, Huntington, WV, USA

**Keywords:** Omega 3, Chemo-sensitization, Chronic lymphocytic leukemia

## Abstract

**Background:**

B-Cell chronic lymphocytic leukemia (CLL) is the most common form of leukemia in the United States. Clinical treatment of CLL is often limited due to drug resistance and severe therapy-induced toxicities. We hypothesized that the omega 3 (n-3) fatty acids, eicosapentaenoic acid (EPA) and/or docosahexaenoic acid (DHA), would increase the sensitivity of malignant B-lymphocytes to anti-cancer drugs doxorubicin, vincristine and/or fludarabine *in vitro* and that increased sensitivity is achieved by alterations in cell-cycle progression leading to growth inhibition and/or enhanced cell death. We further postulate that enhanced sensitivity is dependent on the formation of lipid peroxides and to the generation of reactive oxygen species (ROS).

**Methods:**

In the present study, B-CLL-derived leukemic cell lines EHEB and MEC-2 and the B-Prolymphocytic leukemic-derived (PLL) cell line JVM-2 were tested for *in vitro* sensitivity against doxorubicin, vincristine or fludarabine in the presence or absence of vehicle, arachidonic acid (omega 6), EPA or DHA. Cell cycle analysis and Annexin-V assays were performed to determine cell cycle progression and % apoptotic cells, respectively. Assays for malondialdehyde, a measure of lipid peroxidation, and DCF fluorescence assays, a measure of intracellular ROS, were performed to determine if enhanced sensitivity of cells to the drugs by n-3 was dependent on the formation of ROS.

**Results:**

Our results indicated that: 1) EPA and DHA differentially sensitized B-leukemic cell lines EHEB, JVM-2 and MEC-2 to doxorubicin, vincristine and fludarabine *in vitro*; *2)* n-3 alone and with drug treatment increased cell death and induced G2/M arrest in a cell-type specific manner; 3) lipid peroxidation increased in the presence of n-3; 4) there was higher lipid peroxidation in MEC-2 cells in presence of DHA and doxorubicin than with either alone; 5) n-3 increased generation of ROS in MEC-2, and 6) the addition of vitamin-E abrogated the increase in ROS generation and chemo-sensitivity of MEC-2 to doxorubicin by DHA.

**Conclusion:**

N-3’s are promising chemo-sensitizing agents for the treatment of CLL. Selective enhancement of chemo-sensitivity of EHEB, JVM-2 and MEC-2 to drugs by n-3 that is not dependent on increased lipid peroxidation and ROS generation indicates alternative mechanisms by which n-3 enhances chemo-sensitivity.

## Introduction

B-Cell chronic lymphocytic leukemia (CLL) is the most common form of leukemia in the United States [1]. CLL is a disease of the elderly with two thirds of patients being over 65 years of age at time of diagnosis [1]. CLL remains largely incurable outside of allogeneic transplantation [1]. Despite the success of current treatments such as fludarabine, many patients develop drug resistance and disease relapse [2]. As such, clinical treatment of CLL is often hindered by drug resistance and the non-selectivity of most drugs [3]. Additionally, treatment options for CLL patients who require aggressive treatment are limited due to significant side-effect profiles which are often too toxic for the elderly or those with comorbidities [1]. Given the age group of patients diagnosed with CLL, a therapeutic intervention that can increase the sensitivity of CLL cells to chemotherapy without causing additional adverse effects would be clinically beneficial.

Omega 3 and omega 6 polyunsaturated fatty acids (PUFAs) are essential fatty acids (FAs) which must be obtained from diet. Long chain omega 3 fatty acids (eicosapentaenoic acid (EPA) and docosahexaenoic acid (DHA)) are primarily found in fish oils [4]. The omega 6 fatty acid, arachidonic acid (AA), is primarily found in the meat of animals that consumed corn or soybeans. The ratio of omega 3 FAs to omega 6 FAs in the average western diet is heavily weighted in favor of omega 6 [5,6]. Omega 3 fatty acids have consistently been shown to enhance sensitivity of various solid tumor cells to chemotherapy *in vitro*[7,8] and *in* vivo [9-11]. However, it has not been shown whether n-3 can enhance the sensitivity of CLL to anti-cancer drugs.

Previous studies performed by our group have shown that consumption of an omega 3 supplement, predominantly composed of EPA and DHA, increased the sensitivity of malignant B lymphocytes isolated from patients with early CLL (RAI stages 0, 1) to doxorubicin in an *in vitro* assay [12]*.* These findings prompted us to further evaluate the potential use of omega 3 as a chemo-sensitizing agent for the treatment of CLL. The primary objective of this study was to determine whether EPA and/or DHA could increase the sensitivity of malignant B-lymphocytes to doxorubicin, vincristine and/or fludarabine *in vitro.* Secondary objectives were to elucidate potential mechanism(s) by which n-3 enhance chemo-sensitivity. We hypothesized that EPA and/or DHA would increase the sensitivity of malignant B-lymphocytes to doxorubicin, vincristine and fludarabine *in vitro* and that enhanced sensitivity is mediated by alterations in cell cycle progression leading to enhanced growth inhibition and/or enhanced cell death. We further postulate that increased chemo-sensitivity is dependent, in part, on the formation of lipid peroxides, and the generation of reactive oxygen species (ROS).

In this study we assayed for: 1) fatty acid lipid composition, 2) *in vitro* sensitivity of B-CLL-derived cell lines EHEB, and MEC-2 and B-Prolymphocytic-derived (PLL) cell line JVM-2 against doxorubicin, vincristine and fludarabine in the presence of vehicle (no added FA), AA, EPA or DHA, 3) % of apoptotic cells, 4) cell cycle distribution, 5) generation of intracellular reactive oxygen species (ROS), and 6) levels of lipid peroxidation.

## Results

### N-3 and N-6 fatty acids induce cell death

**Figure 1:**
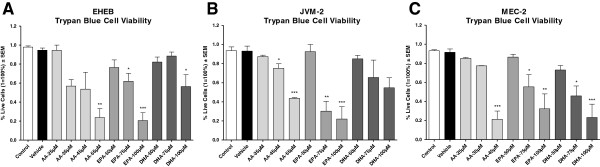
**Determination of optimal FA concentrations.** Figure 1**A**-**C** illustrates the mean % live cells ± SEM of EHEB, JVM-2 and MEC-2 following 72 hour treatment with vehicle, or increasing concentrations of AA, EPA or DHA. Percent (%) live cells was determined after 72 hour treatments by trypan blue exclusion assay. Figure 1A illustrates the mean % live cells ± SEM of EHEB following treatment with vehicle, or increasing concentrations of AA, EPA or DHA. Significant reductions in % live cells were observed at 55 μM AA, 75 μM and 100 μM EPA, and 100 μM DHA as compared to vehicle. Although not statistically significant, concentrations of AA 35 μM and AA 45μM indicated viabilities of 57% and 54%. FA concentrations of AA 25 μM, EPA 50 μM and DHA 75 μM were chosen for the remainder of the study. Figure 1B illustrates the mean% live cells ± SEM of JVM-2 following treatment with vehicle, or increasing concentrations of AA, EPA or DHA. Significant reductions in % live cells were observed at 45 μM and 55 μM AA, and 75 μM and 100 μM EPA as compared to vehicle. Although not statistically significant, concentrations of DHA 75 μM and DHA 100 μM indicated cell viabilities of 65% and 55%. FA concentrations of AA 35 μM, EPA 50 μM and DHA 50 μM were chosen for the remainder of the study. Figure 1C illustrates the mean % live cells ± SEM of MEC-2 following treatment with vehicle, or increasing concentrations of AA, EPA or DHA. Significant reductions in % live cells were observed at 45 μM AA, 75 μM and 100 μM EPA and 75 μM and 100 μM DHA as compared to vehicle. FA concentrations of AA 25 μM, EPA 50 μM and DHA 50 μM were chosen for the remainder of the study. Statistical significant was determined by Multiple Comparison Test using Tukey’s correction. Abbreviations: AA- arachidonic acid, EPA- eicosapentaenoic acid, DHA- docosahexaenoic acid, α = 0.05, * <0.05, ** < 0.01, *** < 0.001.

Figures 1A-C illustrates the % alive cells ± SEM of EHEB, JVM-2 and MEC-2 following treatment with vehicle, or increasing concentrations of AA, EPA and DHA. Cell viability was assessed by Trypan Blue Exclusion assay following treatment for 72 hours. Treatment with AA, EPA or DHA induced dose-responsive reductions in cell viability as compared to vehicle in all three cell lines. We wanted to determine the chemo-sensitizing effects of FA following treatment with concentrations of FA that alone did not induce significant cytotoxicity. Thus, we chose to use concentrations of AA at 25 μM, 35 μM and 25 μM, EPA at 50 μM (all cell lines) and DHA at 75 μM, 50 μM and 50 μM for EHEB, JVM-2 and MEC-2, respectively. The chosen FA concentrations used in this study are clinically achievable [12]. Gas chromatography post 72 hour of FA treatment validated FA incorporation in all cells (Supplementary Data-1).

### EPA and DHA differentially sensitize malignant B-lymphocytes to doxorubicin, vincristine and fludarabine *in vitro*

In our study, we wanted to determine whether FA pre-treatment would synergistically increase the cytotoxic effects (greater effect than the sum effect due to FA and drug individually) of anti-cancer drugs doxorubicin, vincristine or fludarabine on three different B-leukemic cells. Thus, all measurements obtained from the MTT assay following treatment with the anti-cancer drug in the presence of vehicle, or FA were compared to cells treated with vehicle or FA only.

**Figure 2:**
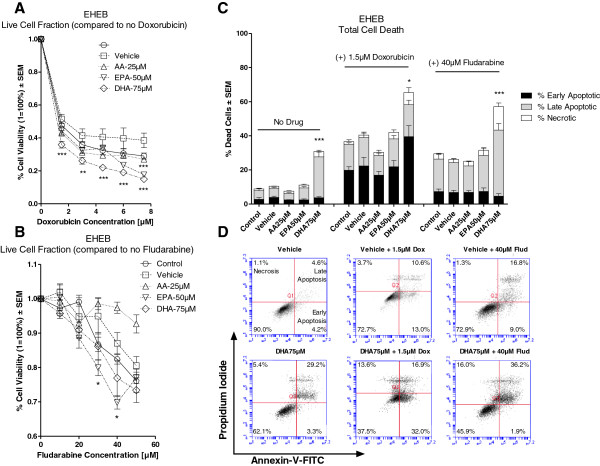
***In vitro *sensitivity of EHEB following treatment with doxorubicin or fludarabine in the presence and absence vehicle, AA, EPA or DHA.** Cell viability was determined by MTT assay. Percent (%) cell death was determined by Annexin-V/Propidium Iodide duel stain with flow cytometry. Figure 2**A** illustrates the % cell viability ± SEM of EHEB to doxorubicin (0–7.5 μM) in the presence or absence of vehicle, AA 25 μM, EPA 50 μM or DHA 75 μM. Cells pre-treated with either EPA or DHA had significantly greater decreases in cell viability as compared to vehicle when treatment with doxorubicin. Figure 2**B** illustrates the % cell viability ± SEM of EHEB to fludarabine (0-50 μM) in the presence or absence of vehicle, AA 25 μM, EPA 50 μM or DHA 75 μM. Pre-treatment of cells with EPA had significantly greater reductions in cell viability as compared to vehicle when treated with 30 μM and 40 μM fludarabine. Figure 2**C** illustrates the % dead cells ± SEM of EHEB following pre-treatment with vehicle, AA 25 μM, EPA 50μM or DHA 75 μM alone and following treatment with 1.5 μM doxorubicin or 40 μM fludarabine. Pre-treatment with DHA alone induced significantly greater cell death as compared to vehicle. Compared to vehicle, cells pre-treated with DHA had significantly higher cell death when treated with doxorubicin or fludarabine. Figure 2**D** provides 2D graphical representations of Annexin-V/PI Plots. ‘Early’ Apoptosis was defined as cells positive for Annexin-V-FITC only. ‘Late’ Apoptosis was defined as cells positive for Annexin-V-FITC and PI. ‘Necrotic’ was defined as cells positive for PI only. Statistical significance was determined by Multiple Comparison Test with Dunnet’s correction (Figure 2**A** and **B**) or Tukey’s correction (Figure 2**C**). α = 0.05, * <0.05, ** < 0.01, *** < 0.001.

Figure 2A illustrates the *in vitro* sensitivity of EHEB to doxorubicin (0–7.5 μM) in the presence or absence of vehicle, AA, EPA or DHA. Compared to vehicle, cell viability was significantly reduced in cells pre-treated with either EPA or DHA but not with AA when treated with doxorubicin.

Figure 2B illustrates the *in vitro* sensitivity of EHEB to fludarabine (0-50 μM) in the presence or absence of vehicle, AA, EPA or DHA. Compared to vehicle, cell viability was significantly reduced in cells pre-treated with EPA when treated with fludarabine (30 μM and 40 μM). However, there was no difference in the sensitivity of EHEB to fludarabine when cells were pre-treated with either AA or DHA. It is interesting to note that AA pretreatment had a non-significant slightly protective effect on EHEB cells treated with fludarabine.

Compared to vehicle, pre-treatment with AA, EPA or DHA did not significantly change the sensitivity of EHEB to vincristine (data not shown).

In our model, an increase in chemo-sensitivity of cells to the drug by FA can be mediated by both enhanced cell death and/or enhanced growth-inhibition. To determine whether the decreases in cell viability seen in the MTT assays were a result of enhanced cell death or of growth-inhibition we performed an Annexin-V assay.

Figure 2C illustrates the % dead EHEB cells (± SEM) in the presence or absence of vehicle, AA, EPA, or DHA alone and after treatment with doxorubicin (1.5 μM) or fludarabine (40 μM). The concentration of doxorubicin (1.5 μM) was chosen as this concentration induced a significant difference in cell viability between FA and vehicle pre-treated cells and because this concentration is clinically achievable [13]. The concentration of fludarabine (40 μM) was chosen as this concentration induced the greatest significant difference in cell viability between EPA and vehicle pre-treated cells (Figure 2B); however, this concentration is ~5-10 times greater than the peak plasma concentration of fludarabine [14]. Compared to vehicle, cells pre-treated with DHA, but without drug, had significantly higher cell death. The addition of doxorubicin or fludarabine to DHA pre-treated cells significantly increased cell death as compared to vehicle and drug treatment. Cell death was mediated predominately through apoptosis (Figure 2C). In cells treated with doxorubicin or fludarabine, pre-treatment with either AA or EPA did not increase cell death as compared to vehicle. Figure 2D displays a graphical 2D representation of Annexin-V/PI plots of EHEB cells pre-treated with either vehicle or DHA and following treatment with doxorubicin or fludarabine.

**Figure 3:**
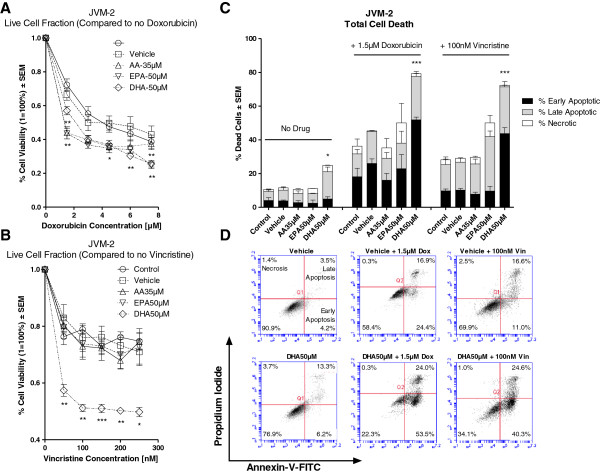
***In vitro *sensitivity of JVM-2 following treatment with doxorubicin or vincristine in the presence and absence vehicle, AA, EPA or DHA.** Cell viability was determined by MTT assay. Percent (%) cell death was determined by Annexin-V/Propidium Iodide duel stain with flow cytometry. Figure 3**A** illustrates the % cell viability ± SEM of JVM-2 to doxorubicin (0–7.5 μM) in the presence or absence of vehicle, AA 35 μM, EPA 50 μM or DHA 50 μM. Cells pre-treated with AA, EPA or DHA induced significantly greater decreases in cell viability as compared to vehicle when treated with doxorubicin. Figure 3**B** illustrates the % cell viability ± SEM of JVM-2 to vincristine (0-250 nM) in the presence or absence of vehicle, AA 35 μM, EPA 50 μM or DHA 50 μM. Compared to vehicle, only cells pre-treated with DHA had significantly greater reductions in cell viability when treatment with vincristine. Figure 3**C** illustrates the % dead cells ± SEM of JVM-2 following pre-treatment with vehicle, AA 35 μM, EPA 50 μM or DHA 50 μM alone and following treatment with 1.5 μM doxorubicin or 100 nM vincristine. Pre-treatment with DHA alone induced significantly greater cell death as compared to vehicle. Compared to vehicle, Cells pre-treated with DHA had significantly greater cell death when treated with doxorubicin or vincristine. Figure 3**D** provides 2D graphical representations of Annexin-V/PI Plots. ‘Early’ Apoptosis was defined as cells positive for Annexin-V-FITC only. ‘Late’ Apoptosis was defined as cells positive for Annexin-V-FITC and PI. ‘Necrotic’ was defined as cells positive for PI only.Statistical significance was determined by Multiple Comparison Test with Dunnet’s correction (Figure 3**A** and **B**) or Tukey’s correction (Figure 3**C**). α = 0.05, * <0.05, ** < 0.01, *** < 0.001.

Figure 3A illustrates the *in vitro* sensitivity of JVM-2 to doxorubicin (0–7.5 μM) in the presence or absence of vehicle, AA, EPA or DHA. Compared to vehicle, all FA pre-treatment significantly decreased cell viability due to doxorubicin treatment.

Figure 3B illustrates the *in vitro* sensitivity of JVM-2 to vincristine (0-250 nM) in the presence or absence of vehicle, AA, EPA or DHA. Compared to vehicle, only DHA pre-treatment significantly decreased cell viability due to vincristine treatment.

Pre-treatment with AA, EPA or DHA did not induce any significant differences in the sensitivity of JVM-2 cells to fludarabine (data not shown).

Figure 3C illustrates the % dead JVM-2 cells (± SEM) in the presence or absence of vehicle, AA, EPA, or DHA alone and following treatment with doxorubicin (1.5 μM) or vincristine (100 nM). The concentration of vincristine (100 nM) was chosen as this concentration induced a significant difference in cell viability between DHA and vehicle pre-treated cells and because this concentration is clinically achievable [15]. Compared to vehicle, pre-treatment with DHA alone induced significant cell death. Pre-treatment with DHA significantly increased cell death due to either doxorubicin or vincristine treatment. Figure 3D displays a graphical 2D representation of Annexin-V/PI plots of JVM-2 cells pre-treated with either vehicle or DHA and following treatment with doxorubicin or vincristine.

**Figure 4:**
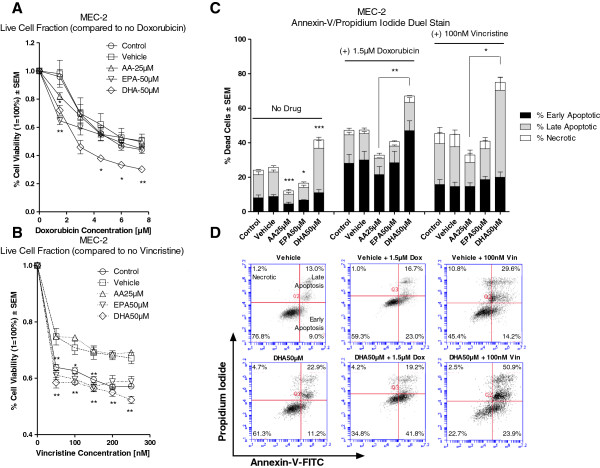
***In vitro *sensitivity of MEC-2 following treatment with doxorubicin or vincristine in the presence and absence vehicle, AA, EPA or DHA.** Cell viability was determined by MTT assay. Percent (%) cell death was determined by Annexin-V/Propidium Iodide duel stain with flow cytometry. Figure 4**A** illustrates the % cell viability ± SEM of MEC-2 to doxorubicin (0–7.5 μM) in the presence or absence of vehicle, AA 25 μM, EPA 50 μM or DHA 50 μM. Cells pre-treated with either EPA or DHA had significantly greater decreases in cell viability as compared to vehicle when treated with doxorubicin. Figure 4**B** illustrates the % cell viability ± SEM of MEC-2 to vincristine (0-250 nM) in the presence or absence of vehicle, AA 25 μM, EPA 50 μM or DHA 50 μM. Cells pre-treated with either EPA or DHA had significantly greater decreases in cell viability as compared to vehicle following treatment with vincristine. Figure 4**C** illustrates the % dead cells ± SEM of MEC-2 following pre-treatment with vehicle, AA 25 μM, EPA 50 μM or DHA 50 μM alone and following treatment with 1.5 μM doxorubicin or 100 nM vincristine. Compared to vehicle, pre-treatment DHA alone induced significantly greater cell death; whereas pre-treatment with either AA or EPA induced significantly lower cell death. Cells pre-treated with DHA had higher cell death, as compared to vehicle, when treated with doxorubicin or vincristine; however, this was only significant when compared against AA pre-treated cells. Figure 4**D** provides 2D graphical representations of Annexin-V/PI Plots. ‘Early’ Apoptosis was defined as cells positive for Annexin-V-FITC only. ‘Late’ Apoptosis was defined as cells positive for Annexin-V-FITC and PI. ‘Necrotic’ was defined as cells positive for PI only. Statistical significance was determined by Multiple Comparison Test with Dunnet’s correction (Figure 4**A** and **B**) or Tukey’s correction (Figure 4**C**). α = 0.05, * <0.05, ** < 0.01, *** < 0.001.

Figure 4A illustrates the *in vitro* sensitivity of MEC-2 to doxorubicin (0–7.5 μM) in the presence or absence of vehicle, AA, EPA or DHA. Compared to vehicle, pre-treatment with either EPA or DHA significantly decreased cell viability due to doxorubicin treatment.

Figure 4B illustrates the *in vitro* sensitivity of MEC-2 to vincristine (0-250 nM) in the presence or absence of vehicle, AA, EPA or DHA. Compared to vehicle, pre-treatment with either EPA or DHA significantly decreased viability of cells treated with vincristine.

Pre-treatment of cells with AA, EPA or DHA did not increase the sensitivity of MEC-2 to fludarabine(data not shown).

Figure 4C illustrates the % dead cells ± SEM of MEC-2 in the presence or absence of vehicle, AA, EPA, or DHA alone and following treatment with doxorubicin (1.5 μM) or vincristine (100 nM). Compared to vehicle, cells pre-treated with DHA alone had significantly higher cell death; whereas cells pre-treated with either AA or EPA had significantly less cell death. The addition of doxorubicin or vincristine to DHA pre-treated cells induced higher cell death as compared to vehicle; however, this was only significant when compared to AA pre-treated cells. Figure 4D displays a graphical 2D representation of Annexin-V/PI plots of MEC-2 cells pre-treated with either vehicle or DHA and following treatment with doxorubicin or vincristine.

### N-3 alone and in combination with anti-cancer drugs induce G2/M arrest

We wanted to determine whether increased chemo-sensitivity by FA was also associated with enhanced growth-inhibition; thus, we performed a cell-cycle analysis. Inhibition of cell-cycle progression leads to growth-inhibition (reduced proliferation).

Table 1 illustrates the mean G1/G2 ratio ± SEM of all three cell lines in the presence of vehicle, AA, EPA or DHA alone and following treatment with 1.5 μM doxorubicin (EHEB, JVM-2, MEC-2), 40 μM fludarabine (EHEB), or 100 nM vincristine (JVM-2, MEC-2). Cell cycle analysis was not performed on EHEB following treatment with vincristine or on JVM-2 and MEC-2 following treatment with fludarabine as there were no significant differences in the *in vitro* sensitivity of these cell lines to these drugs in the presence AA, EPA or DHA as compared to vehicle.

**Table 1:** Pre-treatment with FAs alone and following treatment with doxorubicin, vincristine or fludarabine induces G2/M arrest

**Table 1. Cell cycle analysis: G1/G2 ratio**									
**EHEB**									
	Control ± SEM	Vehicle ± SEM	p-value	AA25 μM ± SEM	p-value	EPA50 μM ± SEM	p-value	DHA75 μM ± SEM	p-value
(-) Drugs	4.1 ± 0.09	4.3 ± 0.04	NS	3.7 ± 0.8	NS	4.0 ± 0.50	NS	4.2 ± 0.20	NS
(+) 1.5 μM Dox	9.2 ± 0.21	9.0 ± 0.27	NS	9.5 ± 0.32	NS	8.9 ± 0.72	NS	5.8 ± 0.77	<0.05
(+) 40 μM Flud	13.3 ± 0.21	15.5 ± 0.04	NS	13.5 ± 0.19	NS	11.6 ± 2.08	NS	4.9 ± 0.22	<0.01
**JVM-2**									
	Control ± SEM	Vehicle ± SEM	p-value	AA35 μM ± SEM	p-value	EPA50 μM ± SEM	p-value	DHA50 μM ± SEM	p-value
(-) Drugs	2.4 ± 0.14	2.4 ± 0.04	NS	2.2 ± 0.06	NS	1.8 ±0.12	NS	1.8 ± 0.09	<0.05
(+) 1.5 μM Dox	2.9 ± 0.16	3.1 ± 0.18	NS	2.6 ± 0.00	NS	2.6 ± 0.07	NS	2.18 ± 0.23	<0.05
(+) 100 nM Vin	1.2 ± 0.00	1.2 ± 0.02	NS	0.9 ± 0.00	<0.05	0.8 ± 0.01	<0.01	0.9 ± 0.06	<0.05
**MEC-2**									
	Control ± SEM	Vehicle ± SEM	p-value	AA25 μM ± SEM	p-value	EPA50 μM ± SEM	p-value	DHA50 μM ± SEM	p-value
(-) Drugs	5.2 ± 0.29	4.8 ± 0.10	NS	4.9 ± 0.37	NS	3.6 ± 0.14	<0.05	2.8 ± 0.28	<0.001
(+) 1.5 μM Dox	3.0 ± 0.11	2.8 ± 0.23	NS	1.5 ± 0.08	<0.001	1.12 ± 0.10	<0.001	1.1 ± 0.15	<0.001
(+) 100 nM Vin	1.2 ± 0.07	1.4 ± 0.19	NS	0.7 ± 0.06	<0.05	0.6 ± 0.04	<0.01	0.9 ± 0.18	NS

Table 1 illustrates the G1/G2 ratios ± SEM of EHEB, JVM-2 and MEC-2 following treatment with 1.5 μM doxorubicin, 100 nM vincristine or 40 μM fludarabine in the presence or absence of vehicle, AA, EPA or DHA. Treatment with DHA alone indicated significantly lower ratios of G1/G2, indicative of G2/M arrest, as compared to vehicle in all three cell lines. Treatment with EPA indicated a significantly lower G1/G2 ratio as compared to vehicle in MEC-2. Pre-treatment with AA, EPA or DHA indicated significantly lower G1/G2 ratios as compared to vehicle following treatment with doxorubicin or vincristine. Only pre-treatment with DHA indicated a significantly lower G1/G2 ratio as compared to vehicle following treatment with fludarabine in EHEB. Statistical significance was determined by Multiple Comparison Test using Tukey’s correction. Abbreviations: Dox: doxorubicin, Vin: Vincristine, Flud: Fludarabine. α = 0.05, NS= not significant.

FA treatment alone: A significantly lower G1/G2 ratio indicates G2/M arrest. Treatment with EPA alone induced a significantly lower G1/G2 ratio, as compared to vehicle in MEC-2 (p: <0.05). Treatment with DHA alone induced a significantly lower G1/G2 ratio as compared to vehicle in JVM-2 and MEC-2 (p: <0.05 and <0.001; respectively). Treatment with AA alone did not induce any significant differences in the G1/G2 ratio as compared to vehicle in EHEB, JVM-2 or MEC-2.

FA plus drug treatment: Cells pre-treated with AA had significantly lower G1/G2 ratios as compared to vehicle when treated with doxorubicin (p: <0.001 in MEC-2) or vincristine (p: <0.05 and <0.05 in JVM-2 and MEC-2, respectively).

Cells pre-treated with EPA had significantly lower G1/G2 ratios as compared to vehicle when treated with doxorubicin (p: <0.001 in MEC-2) or vincristine (p: <0.01 in JVM-2 and MEC-2). Cells pre-treated with DHA had significantly lower G1/G2 ratios as compared to vehicle when treated with doxorubicin (p: <0.05, <0.05, and <0.001 in EHEB, JVM-2 and MEC-2, respectively), fludarabine (p: <0.01 in EHEB), or vincristine (p: <0.05 and <0.001 in JVM-2 and MEC-2, respectively).

### N-3 increases generation of intracellular ROS

To investigate ROS production in response to AA, EPA or DHA alone and following treatment with doxorubicin or fludarabine we used a CM-H_2_DCFDA probe.

**Figure 5:**
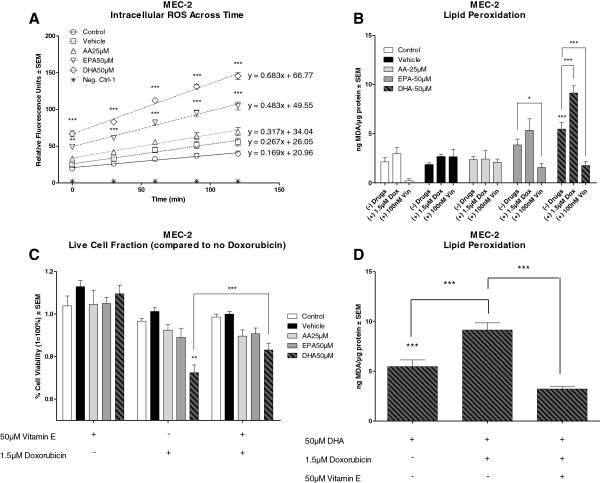
**Chemo-sensitizing capability of DHA is mediated through generation of intracellular ROS and formation of lipid peroxides.** Figure 5**A** illustrates mean relative fluorescence units ± SEM of MEC-2 over the course of 2 hours following 72 hour pre-treatment with vehicle, AA 25 μM, EPA 50 μM or DHA 50 μM. Linear regression analysis indicated a greater slope following treatment with EPA or DHA versus vehicle (0.483 and 0.683 versus 0.267) indicating an increased generation of ROS across time. Figure 5**B** illustrates mean ng MDA/μg protein ± SEM in the presence or absence of vehicle, AA 25 μM, EPA 50 μM or DHA 50 μM alone and following treatment with doxorubicin (1.5 μM) or vincristine (100 nM). Pre-treatment with DHA induced significantly higher levels of MDA as compared to vehicle. The addition of doxorubicin to DHA pre-treated cells induced significantly higher levels of MDA versus either alone. The addition of vincristine to either EPA or DHA pre-treated cells induced significantly lower levels of MDA as compared to n-3 or drug alone. Figure 5**C** illustrates the % cell viability ± SEM of MEC-2 following treatment with 1.5 μM doxorubicin and 50 μM vitamin-E alone and in combination in the presence or absence of vehicle, AA 25 μM, EPA 50 μM or DHA 50 μM. Compared to vehicle, cells pre-treated with DHA had significantly greater reductions in cell viability when treated with doxorubicin. Co-treatment of DHA pre-treated cells with doxorubicin and vitamin-E abrogated the enhanced sensitivity of MEC-2 to doxorubicin by DHA. Figure 5**D** illustrates mean ng MDA/μg protein ± SEM of MEC-2 following pre-treatment with DHA alone and after treatment with 1.5 μM doxorubicin alone and in combination with 50 μM vitamin-E. Co-treatment with doxorubicin and vitamin-E in DHA pre-treated cells indicated significantly lower levels of MDA versus doxorubicin alone. Statistical significance was determined by Multiple Comparison Test with Tukey’s correction. Abbreviations: MDA: malondialdehyde, α = 0.05, * <0.05, ** < 0.01, *** < 0.001.

Figure 5A illustrates mean relative fluorescence units (RFU) ± SEM across time for MEC-2. Of the 3 cell types, ROS were increased only in MEC-2 cells due to pre-treatment with either EPA or DHA. Linear regression analysis indicated that the rate of increase in ROS was significantly greater in DHA pre-treated MEC-2 cells than in vehicle pre-treated cells (Mean slope (RFU)/min: 0.683 versus 0.267, p: <0.01). Similarly, the rate of increase in ROS was greater in the presence of EPA than in vehicle treated MEC-2 cells (Mean slope (RFU)/min: 0.483 versus 0.267, p: 0.08); however this was not statistically significant. Pre-treatment with AA did not induce any differences in the levels of ROS as compared to vehicle.

There were no differences in levels of ROS for EHEB or JVM-2 in the presence of AA, EPA or DHA as compared to vehicle (data not shown). Pre-treatment with vehicle, AA, EPA or DHA followed by treatment with doxorubicin or fludarabine did not induce any significant changes in levels of ROS as compared to vehicle or FA alone in any of the cell lines (data not shown). ROS production in the presence of vincristine was not performed.

### N-3 increases lipid peroxidation

To investigate the formation of lipid peroxides in response to AA, EPA or DHA treatment alone and with doxorubicin, fludarabine or vincristine, levels of thiobarbituric acid reactive substances (TBARS, byproducts of lipid peroxidation) were evaluated and compared to a malondialdehyde (MDA, (a TBARS)) standard curve. Figure 5B illustrates the mean ng MDA/μg of protein ± SEM of MEC-2 treated in the presence of vehicle, AA, EPA or DHA alone and following treatment with 1.5 μM doxorubicin or 100 nM vincristine. Pre-treatment of MEC-2 cells with DHA alone induced significantly higher levels of TBARs than did vehicle. Only DHA pre-treatment induced significantly higher levels of TBARs in doxorubicin treated cells. Cells pre-treated with either EPA or DHA had significantly lower levels of TBARs when treated with vincristine as compared to the FA alone. Analysis of TBARs levels following treatment with fludarabine were not performed as no statistical differences were found in the *in vitro* sensitivity trials. EHEB and JVM-2 had similar trends in levels of TBARs following n-3 pre-treatment as observed in MEC-2; however, the drug treatment did not induce any significant differences as compared to vehicle or n-3 alone (data not shown).

### Vitamin E abrogates enhanced sensitization of MEC-2 to doxorubicin by DHA

To validate that the ability of n-3 to sensitize malignant B-lymphocytes to doxorubicin is dependent on the formation of toxic lipid peroxides, we tested the *in vitro* sensitivity of MEC-2 in the presence of vehicle, AA, EPA or DHA alone and following treatment with 1.5 μM doxorubicin alone or in combination with 50 μM vitamin E. Figure 5C illustrates the *in vitro* sensitivity of MEC-2 to 1.5 μM doxorubicin and 50 μM vitamin E alone and in combination in the presence or absence of vehicle, AA, EPA or DHA. Compared to vehicle, DHA pre-treated cells had significantly greater reductions in viability when treated with doxorubicin. The addition of vitamin E abrogated the enhanced sensitization of MEC-2 to doxorubicin by DHA. In agreement with the *in vitro* sensitivity trial, co-treatment of DHA pre-treated cells with doxorubicin and vitamin E induced significant reductions in the levels of TBARs as compared to doxorubicin alone (Figure 5D).

## Discussion

Chronic lymphocytic leukemia is the most common form of adult leukemia in the western world [1]. Clinical treatment of CLL is often limited due to drug resistance and severe toxicities associated with chemotherapy [1-3]. A therapeutic intervention that could enhance the sensitivity of CLL cells to anti-cancer drugs without causing additional adverse effects would be clinically beneficial.

Omega 3 fatty acids have consistently been shown to enhance the sensitivity of various solid tumor cells to chemotherapy *in vitro*[7,8] and *in vivo*[9-11]. However, this has not been shown in CLL. Previous results from our group indicated that consumption of an n-3 supplement enhanced the sensitivity of lymphocytes isolated from patients with early stage (Rai 0,1) CLL to doxorubicin in an *in vitro* assay [12]. These findings prompted us to further evaluate the potential use of n-3 as chemo-sensitizing agents for the treatment of CLL.

The primary purpose of this study was to illustrate that pre-treatment of B-CLL-and B-PLL-derived cells with n-3 increases the sensitivity of cells to actively used chemotherapeutic drugs: doxorubicin and vincristine, components of the CHOP (cyclophosphamide, doxorubicin, vincristine and prednisone) regimen [16,17], or fludarabine, a commonly used first-line treatment option for CLL [1,2]. Rather than testing combination therapies, we evaluated the ability of n-3 to enhance the sensitivity of malignant B-lymphocytes to single-arm treatments. Secondary objectives were to elucidate potential mechanism(s) by which n-3 enhanced chemo-sensitivity.

Although designated as a single disease, CLL is characterized by biological and clinical heterogeneity. For these reasons, we particularly wanted to demonstrate that the chemo-sensitizing effects of n-3 were not limited to one specific cell sub-type (cell line). Rather we wanted to demonstrate that the chemo-sensitizing effects of n-3 would be seen in multiple cell-types. Thus, each cell line could be viewed as a distinct case of CLL.

For the purposes of this study, we used the highest concentrations FAs that alone did not induce significant cytotoxicity (Figures 1A-C). Our results indicated that clinically achievable concentrations of EPA and DHA generally, but not equally, sensitized the B-leukemic cells to the drugs. Only JVM-2 cells were sensitized to doxorubicin (1.5 μM) when cells were pre-treated with AA (Figure 3A), indicating that the chemo-sensitizing capabilities of FAs are more likely to be found amongst n-3 fatty acids than n-6 fatty acids. This is an important consideration. The western diet is heavily favored towards n-6 FA with little to no n-3 FA intake [5,6]. Omega 3 and n-6 FAs compete with each other for incorporation into the cell [5,6]. The addition of n-3 as an augment to therapy may, therefore, provide clinical benefit to the patient receiving therapy. We are currently conducting a clinical trial to determine if we will see the same chemo-sensitizing capabilities of n-3 on lymphocytes isolated from patients with CLL.

We have illustrated that pre-treatment with n-3 increased the sensitivity of B-CLL- and B-PLL-derived cells to three actively used chemo-therapeutic drugs. While doxorubicin, vincristine and fludarabine have different mechanisms by which they exert their cytotoxic effects, all three drugs can induce cell death and/or growth-inhibition. Thus, increasing the sensitivity of cells to the drug is not a function limited to increased cell death, but can also be mediated through increased growth-inhibition (reduced proliferation). Both cell death and/or growth-inhibition leads to a decrease in numbers of viable cells in culture. For these reasons, we performed Annexin-V assays, as a measure of cellular death, and cell cycle analyses, as an indirect measure of growth (proliferation). Increased cell death and/or increased growth-inhibition are clinically relevant and would provide benefit to the patient.

Collectively, our results indicated that pre-treatment with DHA, as compared to vehicle, enhanced cell death due to doxorubicin in all three cell lines, vincristine in two (JVM-2 and MEC-2) of the three cell lines, and fludarabine in one (EHEB) of the three cell lines. Increased cell death is clinically beneficial and would improve the outcome of the patient receiving therapy.

Noteworthy, MEC-2, which harbors a p53 mutation, showed enhanced cell death due to vincristine or doxorubicin when pre-treated with DHA as compared to vehicle. This is an important observation. The loss of short arm p13 of chromosome 17, which disrupts the p53 tumor suppressor gene, is found in approximately 5-10% of all CLL patients and is associated with particularly poor prognosis and chemorefractoriness [18]. N-3 may provide a beneficial augment to the treatment of chemorefractory CLL patients.

We performed cell-cycle analyses to determine whether increased chemo-sensitivity by FA was associated with enhanced growth-inhibition. Previous studies have demonstrated that n-3 treatment alone can induce cell cycle arrest at the G2/M phase [19]. Vincristine is a mitotic inhibitor known to induce cell cycle arrest at the M phase [20]. Similarly, studies have indicated that malignant cells in G2/M arrest are more sensitive to doxorubicin than normal cells [21,22]. For these reasons, we were particularly interested in the ratio of cells in G1 (G0 + G1) and G2 (G2 + M) phases. An increase in the population of cells in the G2 (G2 + M) phase is indicative of G2/M arrest. Thus, an increase in G2 (G2 + M) would result in a lower population of cells in G1 (G0 + G1) and a lower G1/G2 ratio. A decrease in the G1/G2 ratio (indicative of G2/M arrest) would be expected to result in growth-inhibition (reduced proliferation).

Our results illustrate that cells pre-treated with n-3, but without drug, had significantly greater G2/M arrest, indicated by a lower G1/G2 ratio, as compared to vehicle pre-treated cells (Table 1). This demonstrates that n-3 by themselves can potentially slow the growth of malignant B-lymphocytes. This is of considerable interest as we had previously shown that consumption of n-3 decreased the activity of nuclear factor kappa B (NFκB) in isolated lymphocytes of patients with early stage CLL and would be expected to slow the progression of the disease [12]. Studies have shown that inhibition of NFκB activation leads to cell cycle arrest at the G2/M phase aiding to both growth-inhibition and cell death [23,24]. Future studies will be aimed in determining if n-3 can slow the progression and growth of CLL and whether growth-inhibition is mediated through suppression of NFκB activation and G2/M arrest. Slowing the progression of CLL by n-3 FAs could be a therapeutic choice in patients for whom standard chemotherapy is not an option.

The addition of doxorubicin to FA pre-treated cells induced significantly greater G2/M arrest than when cells were not treated with n-3 prior to doxorubicin (Table 1). It is interesting to note that cells pre-treated with either EPA (MEC-2) or DHA (EHEB, JVM-2 and MEC-2) which had significantly greater G2/M arrest due to doxorubicin also showed increased chemo-sensitivity to doxorubicin than did cells pre-treated with vehicle (Figures 2A, 3A and 4A). This suggests that n-3 plus doxorubicin induced greater growth-inhibition than doxorubicin alone. This notion is supported by other investigators who have shown that cells in G2/M arrest are more sensitive to doxorubicin as compared to normal cells [21,22] and, importantly, that enhanced sensitivity of cells in G2/M arrest to doxorubicin was mediated through both growth-inhibition and apoptosis [22].

Similarly, the addition of vincristine (JVM-2 and MEC-2) or fludarabine (EHEB) to cells pre-treated with certain FAs (all FAs in JVM-2, AA and EPA in MEC-2, DHA in EHEB) had significantly greater G2/M arrest as compared to vehicle pre-treated cells (Table 1). However, there was no association between the increase in chemo-sensitivity of cells to vincristine (JVM-2 and MEC-2) or fludarabine (EHEB) by FA and the increase in G2/M arrest.

Numerous pre-clinical studies have demonstrated that enhanced chemo-sensitization by n-3, particularly DHA, was dependent on the formation of toxic lipid peroxides and generation of ROS [7,11,25-28]. We wanted to determine whether the increase in chemo-sensitivity of cells to the anti-cancer drugs by FA was dependent on the induction of oxidative stress. Our results illustrate that n-3 induced significantly higher levels of intracellular ROS than did vehicle in MEC-2 cells (Figure 5A). Linear regression analysis indicated an increased rate of ROS generation in the presence of either EPA or DHA as compared to vehicle. However, this effect was not enhanced by the addition of any of the anti-cancer drugs. Results also illustrate that treatment with n-3 alone induced higher levels of TBARS, (products of lipid peroxidation), as compared to vehicle in all three cell lines (only MEC-2 is shown, Figure 5B). Only MEC-2 had significantly higher levels of TBARs following treatment with doxorubicin in cells pre-treated with DHA as compared to cells treated with DHA or doxorubicin alone (Figure 5B). The addition of vitamin E, a fat soluble anti-oxidant, abrogated the enhanced sensitivity of MEC-2 to doxorubicin by DHA (Figure 5C) and decreased the levels of TBARS (Figure 5D). The fact that enhanced sensitivity of MEC-2 to doxorubicin by DHA and increased formation of TBARs was abrogated by vitamin E supports the notion that enhanced chemo-sensitivity by DHA is, in part, dependent on the formation of lipid peroxides.

In conclusion, EPA and DHA differentially sensitized B-leukemic cell lines EHEB, JVM-2 and MEC-2 to doxorubicin, vincristine and fludarabine *in vitro*. Enhanced chemo-sensitivity is likely mediated through both increased cellular death as well as growth-inhibition. Our results have shown that enhanced sensitivity is also, in part, dependent on the formation of toxic lipid peroxides. Additional work should be done to elucidate the mechanisms by which n-3 increase chemo-sensitivity. Supplementation of the diet with n-3 fatty acids provides a promising non-toxic approach to not only sensitize CLL cells to anti-cancer drugs but may have independent therapeutic benefit. Importantly, the chemo-sensitizing effects of n-3 do not appear to be limited to a specific cell-type or a specific drug. Increased chemo-sensitivity is clinically beneficial and would be expected to increase drug efficacy, and potentially reduce drug dosage resulting in decreased drug-induced toxicities.

## Materials and methods

### Chemicals

Ninety-five percent pure doxorubicin hydrochloride (Fisher Scientific), and 2-fluoroadenine-9-β-D-arabinofuranoside (Sigma Aldrich) were dissolved in dimethyl sulfoxide (DMSO) to stock solutions and diluted to the working concentrations in cell type specific culture media. Vincristine sulfate salt (Sigma Aldrich) was dissolved in ddH_2_O to stock solutions and diluted to the working concentrations in cell type specific culture media. Vitamin E (α-tocopherol) (Sigma Aldrich) was dissolved in ethanol to stock solutions and diluted to working concentrations in the cell type specific media.

### Cell lines

EHEB (B-CLL), JVM-2 and MEC-2 (B-Prolymphocytic Leukemia) were obtained from Deutsche Sammlung von Mikroorganismen und Zellkulturen (DSMZ). Cells were grown in 1640 RPMI (ATCC) (EHEB and JVM-2) or Iscove’s Modified Dulbecco’s Medium (HyClone, Thermo Scientific) (MEC-2) supplemented with 10% Fetal Bovine Serum (HyClone, Thermo Scientific), 100 units/mL penicillin and 0.1 mg/mL streptomycin. All cell lines were grown in humidified incubator at 37°C and 5% CO_2_.

### Fatty acid treatments

Stock solutions of 100 mM eicosapentaenoic acid (EPA), docosahexaenoic acid (DHA) or arachidonic acid (AA) (Cayman Chemical) in ethanol were made and diluted to the working concentrations in cell type specific culture media. Fatty acid concentrations used represent the concentration of each fatty acid that alone did not induce significant cytotoxicity. One million five hundred thousand (1.5×10^6^) cells per well were seeded in a 6 well plate (Santa Cruz Biotechnology, Inc) and treated with vehicle (ethanol only), AA, EPA or DHA at the stated concentration for 72 hours. Cells were treated for 72 hours with FA to allow adequate time for FA incorporation and to allow for any FA-dependent changes in cellular function. Chosen concentrations of FA are clinically achievable [12]. Post 72 hours, cells were counted with a hemocytometer and prepared for the assays below.

### Lipid composition

Fatty acid composition was assessed by gas chromatography according to our routine techniques [29]. Post 72 hours, cells were washed twice with 1X PBS. Cells were subsequently homogenized in distilled water with 0.1% BHT to prevent fatty acid oxidation. Lipids were extracted with chloroform/methanol, and then methylated. Methylated lipids were separated and identified using gas chromatography as previously published [29]. Fatty acid methyl ester standards (Nu-Chek-Prep, Elysian, MN) were used for peak identification.

### Sensitivity trials

Cell counts were performed and viability was determined by Trypan Blue Exclusion assay following 72 hour fatty acid treatments (FA). Approximately 1x10^5^ living cells/well were seeded in triplicate into a round bottom 96 well plate (CELLSTAR, Greiner Bio One International AG). Cells were subsequently treated with culture media containing DMSO, H_2_O (solvent controls), doxorubicin (0–7.5 μM), vincristine (0-250 nM) or fludarabine (0-50 μM) without FA for 20 hours (doxorubicin) or 24 hours (vincristine, fludarabine). Cells were treated in the presence or absence of 50 μM vitamin E alone and in combination with doxorubicin (1.5 μM) after 72 hour FA pre-treatment for Vitamin E rescue trials. Cell viability was determined using colorimetric MTT 3-(4,5-dimethylthiazol-2-yl)-2,5-diphenyltetrazolium bromide) (CALBIOCHEM, EMD MILLIPORE) assay. Cell viability was assessed by measuring the intensity of precipitate formed, relative to control specimens. Absorption was measured using a SpectraMax M2 (Molecular Devices, Sunnyvale, CA) spectrophotometer at 570 nm. All measurements obtained from the MTT assay following treatment with the anti-cancer drugs in the presence of vehicle, AA, EPA or DHA were compared to cells treated with the vehicle or FA alone. MTT assays were performed in technical and biological triplicate.

### Measurement of apoptosis by annexin-V/propidium iodide duel stain

Apoptosis was measured by duel stain immunofluorescence flow cytometry. Briefly, post 72 hour FA treatments, cell counts were performed and approximately 5 × 10^5^ cells were treated in the presence of DMSO, H_2_O (solvent controls), doxorubicin (1.5 μM), vincristine (100nM) or fludarabine (40 μM) as previously described under sensitivity trials. Cells were washed twice with cold 1X PBS and subsequently incubated for 15 minutes in the dark in 100 μL of Annexin-V binding buffer (0.01 M HEPES, 0.14 M NaCl, 2.5 mM CaCl_2_), 1 μL Annexin-V Alexa Fluor 488-Conjugate (Invitrogen), and 10 μg/mL propidium iodide (Sigma Aldrich). Cells were analyzed using an Accuri Flow Cytometer. ‘Early’ Apoptosis was defined as cells positive for Annexin-V-FITC only. ‘Late’ Apoptosis was defined as cells positive for Annexin-V-FITC and Propidium Iodide (PI). ‘Necrotic’ was defined as cells positive for PI only (Equation 1). Total% cell death was defined as the sum population of cells in early apoptosis, late apoptosis and necrosis. Annexin-V assays were performed in biological triplicate.

$$\begin{array}{l} \text{Equation}\;1:\text{Total}\;\%\;\text{Cell}\;\text{Death}\\ \;=(\%\;\text{Necrotic})+(\%\;\text{Late}\;\text{Apoptosis})\\ \;+\;(\%\;\text{Early}\;\text{Apoptosis}) \end{array}$$

### Cell cycle analysis

Post 72 hour FA treatments, cells were counted and approximately 1 × 10^6^ cells were treated in the presence of DMSO, H_2_O (solvent controls), doxorubicin (1.5 μM), vincristine (100nM) or fludarabine (40 μM) as previously described under sensitivity trials. Cells were subsequently washed twice with cold 1X PBS and resuspended in DNA staining buffer containing 0.2% Triton X-100, 0.2% Na_3_-Citrate, 30 μg/mL RNase and 20 μg/mL propidium iodide (Sigma Aldrich) or DNA staining buffer without propidium iodide to serve as negative controls. Cells were incubated for 30 minutes in the dark at room temperature and subsequently analyzed using an Accuri Flow Cytometer. Cell cycle analyses were performed in biological triplicate. Calculation of G1/G2 ratio is described in equation 2.

$$\begin{array}{l} \text{Equation}\;2:G1/G1\;\text{Ratio}\\ =(\text{\#}\;\text{of}\;\text{events}\;\text{in}\;G0\;+\;G1)/(\text{\#}\;\text{of}\;\text{events}\;\text{in}\;G2\;+\;M) \end{array}$$

### Lipid peroxidation

Lipid peroxidation was measured by means of thiobarbituric acid reactive substances (TBARS) assay. Briefly, approximately 1 to 1.5 × 10^6^ cells were collected in 600 μL 1X PBS post 72 hour fatty acid treatments as described under fatty acid treatments and after doxorubicin (1.5 μM) or vincristine (100 nM) or fludarabine (40 μM)alone or in combination with 50 μM vitamin E (doxorubicin only). Cells were sonicated 2X on 10 second intervals at 40 V setting over ice using a Fisher Scientific Sonic Dismembrator. TBARS assay (Cayman Chemical), in biological triplicate, was performed according to protocol and reported as ng of malondialdehyde/μg of protein.

### Intracellular ROS generation

Levels of intracellular ROS were determined using 5-(and-6-)chloromethyl-2’,7’-dichlorodihydro-fluorescein diacetate, acetyl ester (CM-H_2_DCFDA) (Invitrogen). Briefly, post 72 hour fatty acid treatments, one hundred thousand (10^5^) live cells were seeded in triplicate into a round bottom 96 well plate (CELLSTAR, Greiner Bio One International AG). Cells were washed twice with 1X Dulbecco’s PBS (DPBS) (GIBCO, Invitrogen) and incubated for 60 minutes in the presence or absence of 10 μM CM-H_2_DCFDA in Dulbecco’s Modified Eagles Medium (DMEM, Thermo Scientific) (SIGMA) without FBS containing 100 units/mL pencillin, 0.1 mg/mL streptomycin. Post 60 minute incubation, cells were washed 2X with 1X DPBS and treated in the presence or absence of DMSO, doxorubicin (1.5 μM), or fludarabine (40 μM) where after cell suspensions were transferred to a 96 well flat bottom black well plate (VWR International, LLC). Fluorescence was measured every 10 minutes for 2 hours using SpectraMax M2 spectrophotometer (Molecular Devices, Sunnyvale, CA) at 480 nm excitation 530 nm emission. Assays for intracellular ROS generation were performed in technical triplicates and biological duplicates.

### Statistical analysis

Prism^©^ software (Graphpad, Inc., La Jolla, CA) was used for statistical analysis of numeric data by Multiple Comparison using appropriate Post-Hoc Test and for linear regression analyses. Prism^©^ software (Graphpad, Inc., La Jolla, CA) was used for preparation of graphs. Statistical significance on Annexin-V assays is based on total% cell death as described in equation 1.

## Competing interests

The authors declare that they have no competing interests.

## Authors’ contributions

JFF wrote the manuscript, performed all assays and cell culture experiments, analyzed and interpreted data and designed research and received funding with WEH as mentor. WEH critically analyzed the manuscript and performed final approval of manuscript. All authors read and approved the final manuscript.
